# Effect of replacing soybean meal with cottonseed meal on growth, hematology, antioxidant enzymes activity and expression for juvenile grass carp, *Ctenopharyngodon idellus*

**DOI:** 10.1007/s10695-011-9590-0

**Published:** 2012-02-02

**Authors:** Qingmei Zheng, Xiaobo Wen, Chunyan Han, Haobo Li, Xiaohui Xie

**Affiliations:** 1 College of Animal Sciences, South China Agricultural University, Guangzhou, 510642 China; 2 College of Life Sciences, Jiaying University, Meizhou, 514015 China

**Keywords:** Grass carp (*Ctenopharyngodon idellus*), Soybean meal, Cottonseed meal, Growth, Antioxidant enzymes, Gene expression

## Abstract

An 8-week feeding trial was conducted to evaluate the effect of replacing soybean meal (SBM) with cottonseed meal (CSM) on growth and health of grass carp. Four isonitrogenous diets containing 0, 16.64, 32.73 and 48.94% of CSM, respectively, as replacements of 0, 35, 68 and 100% of SBM were fed to fish (initial body weight 7.14 ± 0.75 g/fish) in triplicate aquaria twice daily. The results indicated that fish fed diet containing 16.64% CSM as a replacement of 35% of SBM was not affected in weight gain (WG), feed efficiency ratio (FER) and feed conversion ratio (FCR) (*P* > 0.05), while fish fed diets containing higher level of dietary CSM (32.73 and 48.94%) significantly decreased WG and PER and significantly increased FCR (*P* < 0.05). Fish fed diets containing 16.64% of CSM had significantly increased hematocrit (Ht) and hemoglobin (Hb) values compared with fish fed with other diets (*P* < 0.05). The activity of catalase (CAT), glutathione peroxidase (GSH-Px) and superoxide dismutase (SOD), gene expression levels of GSH-Px and CAT, and content of malondialdehyde (MDA) were significantly lower for fish fed diets containing 16.64% CSM compared with fish fed other diets (*P* < 0.05). These results showed 16.64% CSM could be used to replace 35% SBM in the diets of juvenile grass carp and without health impact.

## Introduction

Soybean meal (SBM) is a widely available protein source with relatively abundant digestible protein, high energy contents and good amino acid profile (Hertrampf and Piedad-Pascual 2000). SBM is used as a cost-effective feed ingredient and comprises up to 50% of the diet of freshwater fish species (Yue and Zhou 2008). However, the anti-nutritional factors present in SBM such as lectins, protease inhibitors, antigenic compounds and so on tend to thwart the fish’s growth and effect of pathological changes on its intestinal mucosa (Francis et al. 2001). Once the SBM level exceeded 50% in the feed, this could reinforce the mucous membrane inflammation and the damage done to the intestinal mucous membrane (Van den Ingh et al. 1991; Burrells et al*.* 1999). So, it is very necessary to find other plant protein sources to partially or totally replace SBM to solve this problem.

Cottonseed meal (CSM), which generally costs less than both fish meal and SBM, would be beneficial in reducing feed costs for commercial fish farming and ensuring sustainability of the enterprises (Luo et al. 2006). More importantly, CSM contains high levels of proteins (Forster and Cahloun 1995) and is palatable to fish (Robinson and Li 1995). The use of cottonseed proteins as a dietary protein has been examined for many commercial important fish species, such as rainbow trout *Oncorhynchus mykiss* (Cheng and Hardy 2002; Lee et al. 2002; Rinchard et al. 2003), channel catfish *Ictalurus punctatus* (Robinson and Li 1994; Barros et al. 2002), tilapia (El-Sayed 1990; El-Sayed 1999; Mbahinzireki et al*.* 2001; Rinchard et al. 2002; Yue and Zhou 2008), parrot fish *Oplegnathus fasciatus* (Lim and Lee 2009) and sunshine bass *Morone chrysops ♀*×*M. saxatilis ♂* (Rawles and Gatlin 2000). However, the major problem associated with the use of CSM is the toxicity of free gossypol. Free gossypol, when present in large quantity in the diet, could cause unfavorable physiological effects on fish such as depressing growth performance (Barros et al. 2002; Rinchard et al. 2003), decreasing values of hematocrit and hemoglobin (Dabrowski et al. 2001; Yue and Zhou 2008) and reproductive performance (Blom et al. 2001; Rinchard et al. 2003). On the other hand, gossypol was also reported to be a strong natural antioxidant (Bickford et al. 1954) and had received much attention due to its biological activities (Yildirim et al. 2003). CSM and free gossypol were found to improve certain immune responses and disease resistance of channel catfish (Barros et al. 2002; Yildirim et al. 2003).

All animals including fish have immune systems to combat reactive oxygen species (ROS) and thus maintain health and prevent oxidation-induced lesions (Jacob 1995). Due to this, some compounds of these systems are used as labeling of detriment. ROS have been considered as the major mediators of oxygen cytotoxicity (Buetler et al. 2004). These systems include various antioxidant compounds, principally among them being dietary tocopherol, vitamin C and antioxidant defence enzymes (Burton 1990; Buettner 1993). The enzymes include radical-scavenging enzymes such as catalase (CAT) (EC 1.11.1.6) and superoxide dismutase (SOD) (EC 1.15.1.1) acting on hydrogen peroxide (H_2_O_2_) and superoxide (O_2_^−^), respectively, and glutathione peroxidase (GSH-Px) (EC 1.11.1.9), which scavanges H_2_O_2_ and lipid hydroperoxides (Winston and Di Giulio 1991; Halliwell and Gutteridge 1996). These antioxidant enzymes also scavenge free radicals produced by external stimuli (Johnson 2002). Some studies had demonstrated that the antioxidant enzymes were stress- and immune-response biomarkers quantified at enzyme activity and the gene transcript level to evaluate the health impact of animal including fish (Sagstad et al. 2007; Tovar-Ramírez et al. 2010). Besides antioxidant enzymes, malondialdehyde (MDA) was an important indicator of oxidative stress, which was one of the final products of polyunsaturated fatty acids peroxidation in the cells. MDA is known as a marker of oxidative stress and the antioxidant status (Gawel et al. 2004).

Grass carp (*Ctenopharyngodon idellus*) is one of the most important species in the long history of fish farming in China. For the past few years, there have been growing concerns in the business of fish cultivation over minimizing the feed cost, but little regard for anti-nutritional factors present in ingredients, which could affect the physiological status of fish. The objective of the present study is to evaluate whether CSM can be used as a partial or total replacement of SBM without affecting growth and health in grass carp. In this study, an 8-week feeding trial was conducted to evaluate the growth, hematological index, content of MDA, and activity and gene expression of the main antioxidant enzymes in grass carp.

## Materials and methods

### Experimental diets

Four experimental diets containing 0, 16.64, 32.73 and 48.94% of CSM, respectively, as replacements of 0, 35, 68 and 100% of SBM (designated as CSM0, CSM35, CSM68 and CSM100, respectively) on an equal nitrogen basis. The dietary formulation, proximate composition and free gossypol content are presented in Table 1. All diets were formulated to be isonitrogenous (~35% crude protein) and isocaloric. Since SBM and CSM contain different levels of crude fat, the level of soybean oil was adjusted to keep lipid and energy constant in all treatments. All ingredients were ground to pass through a 60 mesh screen and thoroughly mixed before adding water and lipid for final thorough mixture. Then, 2.0-mm-diameter pellets were cold-extruded, air-dried to about 10% moisture, sealed in vacuum-packed bags and frozen (−20°C) for feeding.

**Table 1:** Formulation and proximate analysis of the experimental diets (% dry matter)

Content (%)	Experimental diets			
	CSM0	CSM35	CSM68	CSM100
Ingredients				
Soybean meal	48.00	31.80	16.00	0.00
Cottonseed meal	0.00	16.64	32.73	48.94
Fish meal	10.00	10.00	10.00	10.00
Wheat flour	31.20	31.20	31.20	31.20
Soybean oil	2.60	2.80	3.10	3.30
Dicalcium phosphate	2.00	2.00	2.00	2.00
Mineral premix^a^	0.50	0.50	0.50	0.50
Vitamin premix^b^	0.50	0.50	0.50	0.50
Chloride choline	0.20	0.20	0.20	0.20
Bentonite	3.00	2.36	1.77	1.36
Limestone	2.00	2.00	2.00	2.00
Proximate composition				
Moisture	9.39 ± 0.23	9.27 ± 0.13	8.97 ± 0.04	8.93 ± 0.18
Crude protein	35.29 ± 1.39	35.85 ± 2.20	35.21 ± 1.16	35.86 ± 1.76
Crude lipid	5.42 ± 0.08	5.28 ± 0.146	5.54 ± 0.06	5.48 ± 0.12
Ash	14.56 ± 1.12	14.78 ± 0.86	14.22 ± 2.19	15.15 ± 3.23
Free cossypol(mg/kg)	0.00	69.87 ± 5.29	136.54 ± 8.14	205.83 ± 8.17

Data of proximate composition represent the means ± SD (*n* = 3)

^a^Mineral premix provided the follow minerals (mg/kg diet): ZnSO_4_·7H_2_O, 150; FeSO_4_·7H_2_O, 40; MnSO_4_·7H_2_O, 25; GuCl_2_, 3; KI, 5; CoCl_2_·6H_2_O 0.06; Na_2_SeO_3_, 0.08

^b^Vitaminmixture (mg or IU if mentioned/kg diet): retinylacetate,5,000 IU; cholecalci-ferol, 2,000 IU; all-*rac*-a-tocopheryl acetate, 80 IU; myoinositol, 400; vitamin C,50; menadione sodium bisulfite, 10; thiamin, 10; riboflavin, 5; pyridoxine, 10; D-calcium pantothenate, 50; niacin, 120; biotin,1; foli cacid, 5; vitamin B12, 0.05

### Fish and feeding

Juvenile grass carp were obtained from a local grass carp breeding farm affiliated with Meizhou Fishery Research Institute. Prior to the start of the feeding trial, fish were acclimated to the experimental conditions and fed a commercial diet (~35% crude protein, ~5.60% lipid) for 2 weeks. At the beginning of the feeding trial, healthy fish (initial weight 7.14 ± 0.71 g) were weighed and sorted into 0.75 × 0.5 × 0.8 m plastic tanks, with 14 fish/tank. Three replicate groups of fish were used for each diet. Water was continuously aerated to maintain the dissolved oxygen level above saturation. Water quality parameters were monitored daily between 9:00 and 15:00 h (During the feeding trial, temperature ranged from 22.5 to 27.4°C. Total ammonia nitrogen maintained below 0.05 mg/l, and dissolved oxygen maintained higher than 6.0 mg/l). Fish were fed the experimental diets to apparent satiation twice daily (between 07:30–08:30 and 16:30–17:30 h) for 8 weeks. The amount of feed consumed by the fish in each tank was recorded daily, and rations were adjusted according to feed consumed the previous day. All aquaria were cleaned by scrubbing and siphoning of accumulated wastes, and water change rate was 30% every morning before feeding. The amount of feed consumed was recorded daily by calculating the differences in weight of feeds prior to the first and after the last feeding. Fish in all aquaria were counted and weighed collectively every 2 weeks.

### Sample collection

At the end of the 8-week feeding trial, all fish in each tank were individually weighed and counted for the calculation of weight gain (WG), feed conversion ratio (FCR), protein efficiency ratio (PER) and survival rate (SR). Fish were anesthetized with 0.1% tricane methanesulfonate (MS-222; Argent Chemical Laboratories Inc., Redmond, WA, USA). Blood samples were drawn from the caudal vein of 3 fish randomly selected from each tank; these blood samples were considered to be replicates and were used to determine red blood cell (RBC) count, hematocrit (Ht) and hemoglobin (Hb). Liver samples were collected from 5 fish/tank and immediately stored in liquid nitrogen for analysis of enzymes activities and gene expression.

### Analysis of the component in diet

Crude protein, crude lipid, moisture and ash in diets were determined following standard methods (AOAC 1995). Crude protein (N×6.25) was measured using micro-Kjeldahl nitrogen determination method. Crude lipid was determined by the ether-extraction method. Moisture was determined by oven-drying at 105°C until a constant weight was achieved. Ash content was measured after placing the samples in a muffle furnace at 550°C for 24 h. Free gossypol in diets was extracted by 1,3,5-trihydroxybenzene method described by Zheng et al. (2010), where acetone was used as extractant, and ultrasonic wave as method of sample treatment, and the extraction time was 2 h and extraction temperature was 35°C. Then, the extractive containing free gossypol was filtered by filter paper and determined by spectrophotometric analysis method (absorbance at 550 nm)in 722-type spectro photometer. The content of free gossypol in diets was calculated according to the standard curve of gossypol. The blood samples were assayed with automatic biochemistry analyzer in Meizhou People’s Hostpital.

### Enzymatic and MDA’s measurements

Total protein concentrations were measured in homogenates of liver. A Complete Coomassie Brilliant Blue assay kit (Nanjing Jian-cheng Technolgy Co., Catalog no. A045-2, China) was used for protein measurement following the manufacturer’s instructions. Bovine serum albumin was used as standard with which different dilutions were made to generate a standard curve. Tissue sample dilutions were made according to the absorbance values, which were within the limits of the linearity range on the standard curve.

Approximately 0.3 g liver tissue was homogenized in 3 ml isotonic Na chloride, using a glass homogenizer. The homogenates were centrifuged at 2,500 revolutions per minute (rpm) for 10 min at 4°C. The supernatants were diluted to suitable concentration for assay of GSH-Px, SOD and CAT. CAT activity was analyzed with CAT assay kit (Nanjing Jiancheng Technolgy Co., Catalog no. A007, China). One unit (U) CAT was defined as the amount of enzymes needed to eliminate 1 μmol H_2_O_2_/s. Values of CAT activity are expressed as U/mg total protein. SOD activity in liver was analyzed by use of total SOD assay kit (Nanjing Jiancheng Technolgy Co., Catalog no. A001, China) by xanthine oxidase method according to the manufacturer’s instructions. 1 U of SOD was defined as the amount of enzymes needed to inhibit the rate of O_2_^−^ in 1 ml reaction liquor by 50%. Values of SOD activity are expressed as U/mg total protein. GSH-Px activity in liver was analyzed by use of GSH-Px assay kit (Nanjing Jiancheng Technolgy Co., Catalog no. A005, China) according to the manufacturer’s instructions. Using reduced glutathione hormone as a reducing reagent, the GSH-Px enzymes catalyze the reduction of H_2_O_2_ and organic peroxides (R–O–O–H) to water with the corresponding stable alcohol, thus inhibiting the formation of free radicals. Enzyme activity can be decreased by negative feedback from excess substrate. 1 U of GSH-Px was defined as the amount of enzymes needed to decrease reduced GSH 1 μM/min excluding the values produced by non-enzyme reaction. Values of GSH-Px activity are expressed as U/mg total protein. The content of MDA was assayed by TBA (thibabituric acid) method with a MDA assay kit (Nanjing Jiancheng Technolgy Co., Catalog no. A003-1, China). MDA forms a 1:2 adduct with thiobarbituric acid. The MDA–TBA adduct formed from the reaction of MDA in samples with TBA can be measured colorimetrically. TBARS (thiobarbituric acid reactive substances) levels are determined from a MDA equivalence standard.

### RNA extraction and cDNA cloning

A small piece of liver sample (~80 mg) from grass carp was ground to a fine powder in liquid nitrogen with a mortar and pestle. The frozen powder was transferred to a 1.5-ml Eppendorf tube, and 1 ml of Trizol RNA was added. Total RNA was isolated with trizol according to the manufacturer’s instructions. The amounts of RNA in the samples were quantified by spectrophotometry, whereas the quality of the RNA was verified by agarose gel electrophoresis. And the RNA samples were treated with deoxyribonuclease (DNase) (Promega, Catalog no. M6101) according to the manufacturer’s protocol in order to remove any residual DNA in the preparation.

The first-strand cDNA was performed with M-MLV Reverse Transcriptase kit (Promega, Catalog # M1701). 2 μg of total RNA and 2 μl of oligo(dT)_18_ primer (20 pmol/μl) were incubated at 70°C for 5 min to melt secondary structure within the template. Cool the tube immediately on ice for 2 min to prevent secondary structure from reforming. Then spin briefly to collect the solution at the bottom of the tube. Add the following components to the annealed primer/template in the following. M-MLV Reverse Transcriptase (200 U), 5 μl deoxynucleoside triphosphates (10 mM), RNase inhibitor (25 U), 5×reaction buffer and water were added to yield a final volume of 25 μl, and the reaction mixtures were incubated at 42°C for 60 min and at 95°C for 5 min. The cDNA reaction mixtures were diluted 1/10 and stored in −80°C freezer that were served as templates for PCR amplification.

For cloning gene of GSH-Px, SOD and CAT, forward and reverse primers were designed referring to sequence data from GenBank. The primer sequences, referring to the sequence’s accession number, and the reaction parameters of PCR were described in Table 2. First-strand cDNA was reverse transcribed and served as templates for PCR amplification. The PCR amplification was performed with TaKaRa Taq™ kit (TaKaLa, Catalog no. DR001A) according to the manufacturer’s protocol. PCR products with expected size were excised and purified using gel extraction kit (TIANGEN Biotech CO., LTD, DP209, China). The purified DNA fragments were subcloned into the pMD18-T Simple Vector (TaKaLa, Catalog no. D103A) according to the manufacturer’s protocol and transformed into *E. coli* Top10. Positive colonies were identified and served for making standard curve of the real-time PCR.

**Table 2:** Oligonucleotide primer sequences

Gene	Accession number	Forward and reverse primers (5′ to 3′)	Annealing temp (°C)	PCR product size (bp)
SOD	FJ458445	F: GGACCAACCGATAGTGAAAGACAC	57	268
		R: CCTCTATGATTGGAGCAGGACACT		
CAT	FJ560431	F: CCACTTCTGGTCCAGGATGTGGT	62	192
		R: GCGAACAGCGATGGGTGTCGTCT		
GSH-Px	EU828796	F: GGCACAACAGTCAGGGATTACACT	59	223
		R: GGTGGGCGTTCTCACCATTCACT		

### Real-time quantitative PCR

A two-step real-time-PCR method was applied to measure the gene expression levels (mRNA) of antioxidant enzyme genes of GSH-Px, SOD and CAT in liver of fish. RNA isolation and cDNA synthesis were conducted according to the above-mentioned methods. Real-time RT-PCR was performed according to the user manual of real time RNA PCR Kit (TaKaRa, SYBR^®^ Premix Ex Taq™ II, Code DRR041C).

An absolute quantification method was used to determine the gene expression values. For real-time analysis, samples were quantified by comparison with a standard curve generated by amplifying serial fivefold dilutions of a non-diluted cDNA template with the respective primers. Serial diluted plasmids cloned with target fragments were used as standard samples for the establishment of standard curve. Each sample replicated four times.

The real-time PCR step was performed on an ABI 7500 System (Applied Biosystems, Oslo, Norway). The PCR reactions consisted of 45 cycles in a final volume of 20 μl, using 1 μl 20 × SYBR green I, 2 μl cDNA, 1U EX Taq HS DNA polymerase, 4 μl 5 × PCR buffer, 0.5 μl dNTP, 0.5 μl (20 pmol/μl) forward and reserve primers of target gene and water. Reactions were conducted in reaction tubes, beginning with a 60-s hot-start activation of the *Taq* polymerase at 95°C, followed by 45 cycles of 95°C for 10 s, annealing temperature for 10 s and extension 72°C for 15 s, then for 72°C for 5 min and a final solubility curve assay. Quantification results were analyzed by software of ABI 7500 System to export the Ct value and copy density of each sample so as to determine the mRNA abundance of target genes. The data were further analyzed using Microsoft Office Excel 2003.

### Calculations and statistical analysis

The growth parameters were measured by the following equations:

$$ {\text{WG }}\left( \% \right) = 100 \times \left( {{\text{final mean body weight}}-{\text{initial mean body weight}}} \right)/{\text{initial mean body weight}}; $$

$$ {\text{FCR}}\left( {{\text{g}}/{\text{g}}} \right) = {\text{feed consumed}}\,\left( {{\text{g}},{\text{ dry weight}}} \right)/{\text{wet weight gain}}\,\left( {\text{g}} \right); $$

$$ {\text{PER}}\left( {{\text{g}}/{\text{g}}} \right) = {\text{weight gain}}\,\left( {\text{g}} \right)/{\text{protein intake}}\,\left( {\text{g}} \right); $$

$$ {\text{SR}}\left( \% \right) = 100 \times \left( {{\text{final fish number}}/{\text{initial fish number}}} \right). $$

All results were presented by mean value ± standard deviation (mean ± SD). All data were conducted with SPSS 16.0 software (SPSS Inc., USA) for one-way analysis of variance (one-way ANOVA) and Duncan’s multiple comparison, and *P* < 0.05 were considered to be significantly different.

## Results

### Growth and feed utilization

WG, FCR, PER and SR are presented in Table 3. The results indicated that fish fed diet containing 16.64% CSM as a replacement of 35% of SBM were not affected in WG, FCR and PER compared with fish fed the control diets (*P* > 0.05), while fish fed diet containing higher level of dietary CSM (32.73 and 48.94%) significantly decreased WG and PER and increased FCR (*P* < 0.05). SR of fish in all treatments were high (ranging from 95.7 to 100%). But SR significantly decreased in fish of CSM68 and CSM100 at the later stage of the experiment (*P* < 0.05).

**Table 3:** Mean weight gain, feed conversion ratio protein efficiency ratio and survival rate of the grass carp fed diets containing various levels of cottonseed meal for 8 weeks

Item	Experimental diets			
	CSM0	CSM35	CSM68	CSM100
IBW(g/fish)	7.10 ± 0.74	7.17 ± 0.86	7.12 ± 0.64	7.15 ± 0.78
FBW(g/fish)	16.36 ± 1.34^a^	17.03 ± 1.19^a^	15.32 ± 1.25^b^	14.60 ± 0.93^b^
WG(%)	128.28 ± 7.73^a^	129.81 ± 13.17^a^	113.15 ± 8.14^b^	102.61 ± 2.37^b^
FCR (g/g)	1.44 ± 0.12^a^	1.48 ± 0.08^a^	1.63 ± 0.07^b^	1.65 ± 0.11^b^
PER (g/g)	1.97 ± 0.12^a^	1.94 ± 0.09^a^	1.73 ± 0.08^b^	1.72 ± 0.13^b^
SR(%)	100 ± 00^a^	100 ± 00^a^	96.58 ± 1.48^b^	95.73 ± 1.48^b^

Values are means ± SD. Values in the same row with different superscripts are significantly different (*P* < 0.05)

*IBW* initial body weight, *FBW* final body weight

### Hematological index

Values for red RBC, Ht and Hb were affected by dietary levels of CSM (Table 4). No significant differences were observed for the values of RBC as the replacement of SBM with CSM increased from 0 to 68% (*P* > 0.05), but significantly decreased when the replacement level was up to 100%. Ht values significantly increased as the replacement level of SBM with CSM increased from 0 to 35%, however, significantly decreased as the replacement level increased from 35 to 100% (*P* < 0.05). Fish fed diets containing 16.64 and 32.73% of CSM as replacements of 35 and 68% of SBM had improved Hb (*P* < 0.05) compared with fish of other treatments, but total replacement of SBM by CSM significantly decreased Hb (*P* < 0.05).

**Table 4:** Mean blood cell count, hematocrit hemoglobin and mean corpuscular volume of grass carp fed diets containing various levels of cottonseed meal for 8 weeks

Item	Experimental diets			
	CSM0	CSM35	CSM68	CSM100
RBC (×10^12^ l^−1^)	3.13 ± 0.35^b^	3.75 ± 0.21^a^	3.41 ± 0.29^ab^	2.45 ± 0.25^c^
Ht (%)	37.80 ± 0.87^b^	45.90 ± 0.94^a^	37.50 ± 0.63^b^	28.20 ± 0.77^c^
Hb (g l^−1^)	96 ± 2.51^b^	114 ± 6.54^a^	111 ± 4.35^ab^	77 ± 5.83^c^

Values are means ± SD. Values in the same row with different superscripts are significantly different (*P* < 0.05)

### Antioxidant enzymes and MDA

**Fig. 1:**
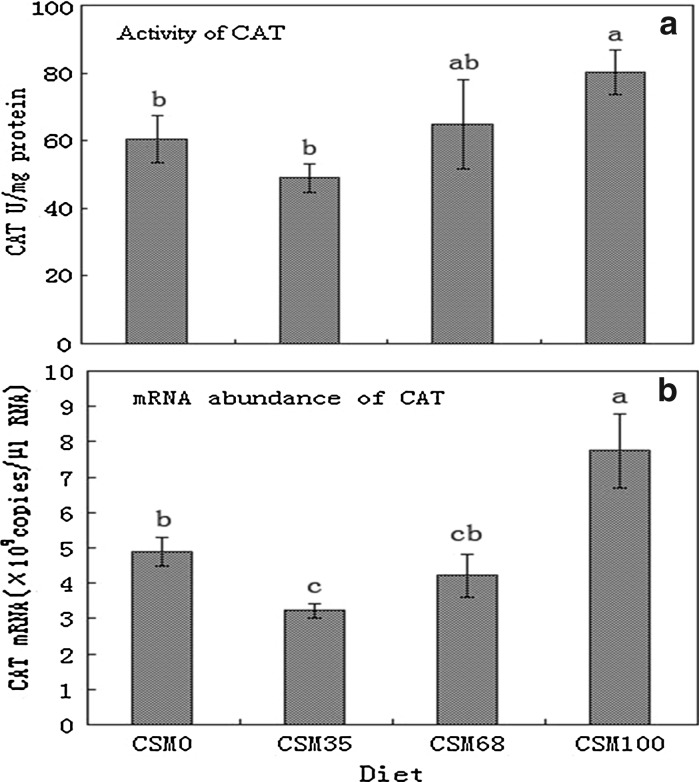
CAT activity (**a**) and CAT normalized expression (**b**) in liver of grass carp fed diets containing various levels of cottonseed meal for 8 weeks. Values are mean ± SD with *different superscripts* are significantly different (*P* < 0.05)

**Fig. 2:**
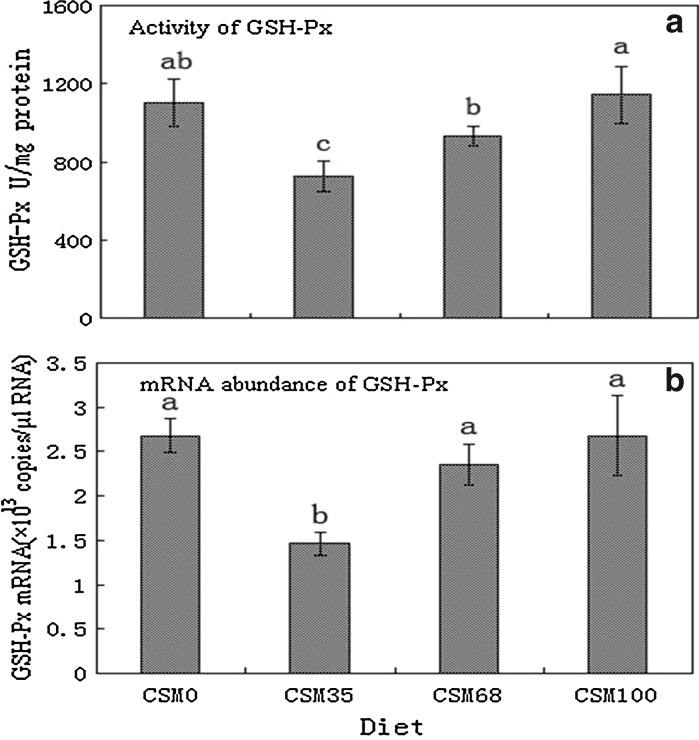
GSH-Px activity (**a**) and GSH-Px normalized expression (**b**) in liver of grass carp fed diets containing various levels of cottonseed meal for 8 weeks. Values are mean ± SD with *different superscripts* are significantly different (*P* < 0.05)

**Fig. 3:**
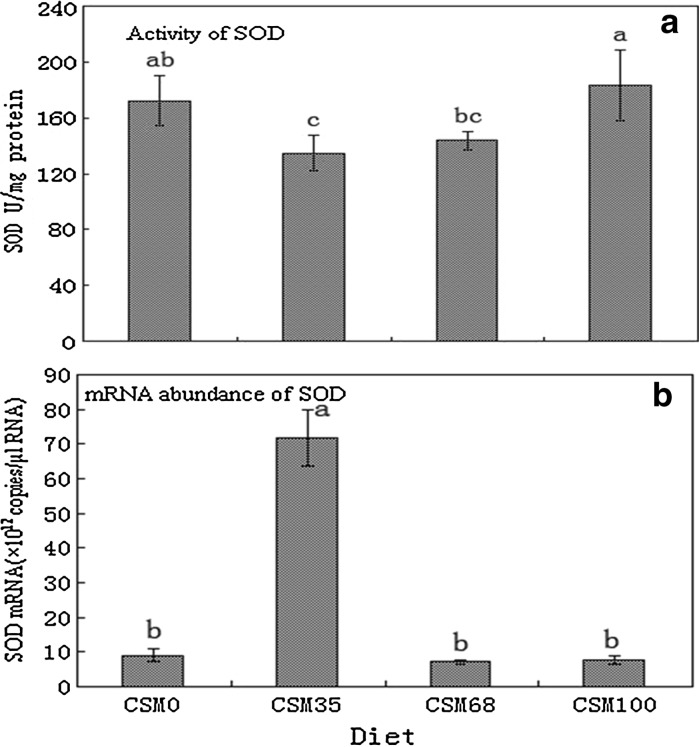
SOD activity (**a**) and SOD normalized expression (**b**) in liver of grass carp fed diets containing various levels of cottonseed meal for 8 weeks. Values are mean ± SD with *different superscripts* are significantly different (*P* < 0.05)

**Fig. 4:**
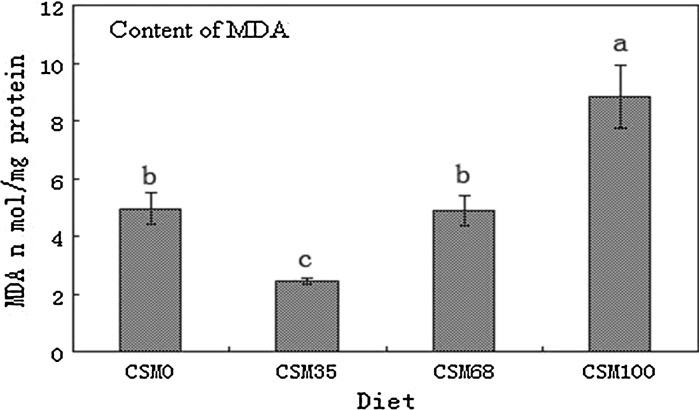
Content of MDA in liver of grass carp fed diets containing various levels of cottonseed meal for 8 weeks. Values are mean ± SD with *different superscripts* are significantly different (*P* < 0.05)

The activity levels and transcriptional responses of antioxidant enzyme genes were readily different in fish fed different diets. The activities of CAT, GSH-Px and SOD significantly increased as the replacement levels increased from 35 to 100% (*P* < 0.05) (Figs. 1a, 2a, 3a), and the activities of CAT of fish fed the highest levels of CSM were significantly higher than those of the control (*P* < 0.05) (Fig. 1a), while the activities of GSH-Px and SOD were similar to those of the control group (*P* > 0.05) (Figs. 2a, 3a). At the same time, the change tendency of MDA was similar to the activities of CAT (Fig. 4). An increase in CAT and GSH-Px mRNA paralleled the increase in the activities of the enzymes (Figs. 1, 2), an increase in SOD gene expression in contrast to a decrease in enzyme activity (Fig. 3).

## Discussion

The results of the present study indicate that CSM protein could be incorporated in diets for juvenile grass carp at a level of 16.64%, which could replace 35% of the protein of SBM, without any adverse effect on growth or health impact while fish fed diet containing higher level of dietary CSM (32.73 and 48.94%) significantly decreased WG and PER and increased FCR. These findings agree with the results obtained by Dorsa et al. (1982), who found that 17.4% glanded CSM (0.49% free gossypol) can be included in channel catfish diets. Lim and Lee (2009) reported that negative effects on growth performance of parrot fish were observed when 30% of fish meal protein was replaced by CSM. Growth depression in animals fed CSM is usually associated with the decrease in the levels of available lysine in diets. Huang et al. (2003) reported that determined by maximum growth rates, the demand quantity of lysine in diets of grass carp was 1.61% of dietary protein. In our study, total dietary lysine was above 6.2% of dietary protein (the lysine content of the ingredients was calculated based on the “Tables of Feed Composition and Nutritive Values in China, 2007”), which was higher than the demand quantity of lysine in feed of grass carp. In addition, most studies revealed that free lysine proved to be ineffective in fish of cyprinidae as dietary protein supplement (Li 1996; Liu et al. 2002; Leng and Wang 2005). Hence, the lysine content in CSM of diets was not the main reason for growth depression in the current study.

Even with added lysine, the free gossypol in CSM could decrease the digestibility of lysine when fish ingested diets with high concentration of gossypol (Liu et al. 2009). Free gossypol is known to bind lysine rendering it less bioavailable. The digestibility ratio of lysine in CSM was only 66% in diets of channel catfish (Wilson et al. 1981). So the amount of dietary CSM that can be used as a protein source for fish seems to depend mainly on its free gossypol and available lysine content of the meal (El-Saidy and Gaber 2004; Luo et al. 2006). With low-gossypol CSM (0.022% free gossypol), a level of 25–30% can be used in channel catfish diets without detrimental effects (Robinson 1991). Yue and Zhou (2008) reported that diets in which 60% of SBM was replaced by 33.76% CSM (28.05 mg free gossypol/kg diets) yielded the best growth and feed utilization. Barros et al. (2002) found that even though lysine was added (total dietary lysine content of 6.2% of dietary protein), total replacement of SBM with CSM (0.122% free gossypol) resulted in reduced WG and feed intake. Liu et al. (2009) suggested that the growth depression in fish could be attributed to the toxicity of free gossypol and decreased digestibility of lysine. In our study, fish fed diets that contained 16.64% CSM (containing 69.87 ± 5.29 mg free gossypol/kg diet) exhibited better weight gain and protein efficiency ratio than those fed 32.73 and 48.94% CSM. This finding indicates that low concentration of free gossypol in CSM could not affect the digestibility of lysine and utilization of the diets. But high concentration of free gossypol could bind lysine rendering it less bioavailable. So the growth depression of fish fed diets containing 68 and 100% of CSM (containing 136.54 ± 8.14 and 205.83 ± 8.17 mg free gossypol/kg diet, respectively) may have been attributed to the toxicity of free gossypol and less available lysine. In addition, free gossypol is a major lipid-soluble substance that binds with blood proteins and cell membranes with high-affinity after ingestion (Royer and Vander Jagt 1983; Reyes and Benos 1988).

The effects of dietary CSM on hematological factors in fish are not consistent in previous studies. Some researchers reported a decrease in Ht and Hb values with an increase in dietary gossypol (Blom et al. 2001; Dabrowski et al. 2001). However, Barros et al*.* (2002) reported that hematological values of juvenile channel catfish were not affected by dietary CSM level of up to 55.0% (671 mg free gossypol/kg diet), even though this level of CSM reduced growth performance. The results obtained in our study were similar to those of El-Saidy and Gaber (2004) and Yue and Zhou (2008) who reported that hematological values were not affected as the dietary CSM levels remained in a reasonable range, but significantly decreased as the dietary CSM levels were too high. Braham and Bressani (1975) suggested this phenomenon could be explained by an adverse effect of gossypol on intestinal iron absorption. Mbahinzireki et al*.* (2001) also suggested that reduced hematological values were one of the most common physiological phenomena of gossypol toxicity in fish.

Antioxidant enzymes were an important indicator of animals’ physical health and reaction in response to external stimuli (Johnson 2002). Some studies indicated the ingredients or nutritional factors in feed could affect the activity and expression of antioxidant enzymes of fish after ingestion (Sitjà-Bobadilla et al. 2005; Fernández-Díaz et al. 2006; Lin et al. 2007; Sagstad et al. 2007; Tovar-Ramírez et al. 2010). Lilleeng et al. (2007) showed that the expression of antioxidant enzymes was significantly up in the intestine of Atlantic cod *Gadus morhua* fed SBM, when compared with the fish fed fish meal. Reyes-Becerril et al. (2008) found that antioxidant enzyme protection of fish was obvious when ROS are generated by xenobiotics or fish exposure to pathogens. The results of various studies reveal that oxidative stress occurs when there is an imbalance between the generation and removal of radical species within an organism. In the present study, fish fed diets containing 16.64% of CSM as replacements of 35% of SBM had significantly decreased the values of most of the antioxidant index compared with fish fed other diets. This finding indicates that oxidative stress did not occur when fish ingested the diets of low levels of CSM (16.64%). But overproduction of ROS was observed in fish fed diets containing the higher levels CSM (32.73 and 48.94%) or control diets, represented by an increase in activity and gene expression levels of antioxidant enzymes. The fluctuation of the antioxidative enzymes might be caused to cope with excessive ROS when fish ingested the diets containing large quantity of gossypol in CSM or some anti-nutritional factors in SBM, for the biochemical compositions of the four groups of diets, were similar. Our results were similar to those obtained by Yildirim et al. (2003) that gossypol adversely affected health status of catfish, even though improved macrophage chemotaxis ratio, serum lysozyme activity and resistance of catfish against *Edwardsiella ictaluri* infection were observed at dietary levels of 900 mg gossypol/kg diet or higher. However, Barros et al*.* (2002) showed gossypol or other compounds present in CSM may have a beneficial effect by improving the immune response and the resistance of juvenile channel catfish against *E. ictaluri* challenge. Thus, further studies need to be conducted to obtain more data on the relationship between gossypol and fish health.

Comparative examination of antioxidant enzymes expressions between mRNA level and protein level could provide valuable information on the mechanism of action in antioxidant enzymes. Nam et al. (2005) demonstrated that the transcriptome might not always be reflected at proteome levels, namely the changes in mRNA might not always be reflected in enzyme activities. In our study, an increase in GSH-Px and CAT mRNA paralleled the increase in the activities of the enzymes while an increase in SOD gene expression was accompanied by a decrease in enzyme activity. The differences in the activity and gene expression patterns of SOD could be attributed to the role post-translational modification played in altering the activity of this enzyme. In addition, the increased mRNA abundance of CAT and GSH-Px and the decreased mRNA abundance of Cu–Zn SOD suggested that there might exist some kind of complementation system between the antioxidant enzymes and oxidative stress. However, further studies are needed to evaluate the effect of gossypol and other compounds in CSM on the health status of fish.

This study has shown that diets in which 35% of SBM was replaced by 16.64% CSM yielded the best growth and health status of fish, while higher levels replacement of SBM (68 and 100%) with CSM reduced the nutritional value of the diet.
